# Frequency of Autoantibodies on Non-Hodgkin Lymphoma

**DOI:** 10.3390/healthcare11152210

**Published:** 2023-08-06

**Authors:** Sonia Guadalupe Barreno-Rocha, Sandra Guzmán-Silahua, Ernesto Germán Cardona-Muñoz, Maria Guadalupe Zavala-Cerna, David Eduardo Muñoz Gaytan, Carlos Riebeling-Navarro, Benjamín Rubio-Jurado, Arnulfo Hernán Nava-Zavala

**Affiliations:** 1 Unidad de Investigación Epidemiológica y en Servicios de Salud, CMNO OOAD, Jalisco, Instituto Mexicano del Seguro Social, Guadalajara 44340, Mexico; dra.barrenorocha@gmail.com (S.G.B.-R.); guzmansilahuasandra@gmail.com (S.G.-S.); david.emgaytan@gmail.com (D.E.M.G.); 2 Programa de Doctorado en Farmacología, Departamento de Fisiología, Centro Universitario Ciencias de la Salud, Universidad de Guadalajara, Guadalajara 44340, Mexico; cameg1@gmail.com; 3 Laboratorio de Investigación en Inmunología, Unidad Académica Ciencias de la Salud, Universidad Autónoma de Guadalajara, Guadalajara 44100, Mexico; maria.cerna@edu.uag.mx; 4 Unidad de Investigación en Epidemiología Clínica, UMAE HP, Centro Médico Nacional SXXI, IMSS, México City 06720, Mexico; criebnava@yahoo.com.mx; 5 Departamento Clínico de Hematología, División Onco-Hematologia, UMAE, Hospital de Especialidades, Centro Médico Nacional de Occidente, Instituto Mexicano del Seguro Social, Guadalajara 44340, Mexico; 6 Programa Internacional de Medicina, Universidad Autónoma de Guadalajara, Zapopan 45129, Mexico; 7 Departamento de Inmunología y Reumatología del Hospital General de Occidente, Secretaría de Salud Jalisco, Guadalajara 45070, Mexico

**Keywords:** autoantibodies, non-Hodgkin lymphoma, clinical outcome, antiphospholipid antibodies, antinuclear antibodies, anticytoplasmic neutrophils antibodies

## Abstract

(1) Background: Non-Hodgkin Lymphoma is a neoplasm that can significantly compromise the immune system, but timely assessment can change the patient outcome. In cancer, the activation of the immune system could lead to the secretion of autoantibodies. (2) Methods: A retrospective cohort study was performed from 2017 to 2019 in patients with Non-Hodgkin Lymphoma diagnosed with a biopsy. (3) Results: We included 39 patients who were newly diagnosed, untreated, and without any autoimmune disease previously reported. Thirty patients had the presence of autoantibodies (antiphospholipid antibodies, anti-cytoplasmic neutrophils antibodies, antinuclear antibodies), and nine were without autoantibodies. There were no statistical differences among groups regarding clinical, demographic, staging, and prognosis characteristics. Also, there were no differences in the outcomes of the patients after finishing chemotherapy and one year after initiating treatment. (4) Conclusions: Further investigations must be conducted regarding an extended panel of autoantibodies because the panel of autoantibodies in this study did not show a relationship between the presence and the clinical outcome of the patients.

## 1. Introduction

Non-Hodgkin Lymphoma (NHL) comprehends a variety of neoplasm with an origin mostly on B cells (up to 90% of them), and the remaining percentage is represented by T cells and Natural Killer (NK) cells [1,2]. The sites with significant development frequency are the lymph nodes, spleen, and bone marrow [1,3]. The states related to immunosuppression or autoimmune diseases are key risk factors related to developing NHL [2,3,4,5]. Regarding the presence of NHL on the global panorama, NHL represents an incidence of 2.8% of new cancer cases and 2.6% of deaths worldwide [6]. In Mexico, the incidence reaches 3.5% of new cases and 3.4% of the deaths related to cancer in the country, also reaching a 5-year prevalence of 19,495 cases [7]. Approximately a third of the cases of NHL correspond to B-cell diffuse large cell lymphoma [1,4].

An early diagnosis is critical for proper treatment [8]; once a patient finishes the treatment, we can assess their response to chemotherapy using The Cheson Criteria [9].

During the immune response to cancer, the immune system can activate the recognition of antigens that would produce autoantibodies [10,11]. The presence of antinuclear and antiphospholipid antibodies [8] has been reported in patients with Hodgkin Lymphoma and Non-Hodgkin Lymphoma [8,12].

For this study, it was hypothesized that the presence of autoantibodies in patients with Non-Hodgkin Lymphoma could be related to the outcome of the patients. Thus, as a study objective, we focus on evaluating the frequency of autoantibodies in patients with Non-Hodgkin Lymphoma who received chemotherapy and the relationship with the outcome of the patients.

## 2. Materials and Methods

This study was performed from 2017 to 2019 in the Hematology Department of the Hospital de Especialidades, Unidad Médica de Alta Especialidad, Centro Médico Nacional de Occidente from the Instituto Mexicano del Seguro Social. It is a retrospective cohort study of consecutive institutional patients with suspected Non-Hodgkin Lymphoma diagnosed with a biopsy. An active search was made for NHL patients who met the selection criteria, and the measurement times for antibodies were before the first administration of chemotherapy. Regarding the measurement items, standardized clinical scales were considered for patients with NHL, taking into account staging scales (Ann Arbor) and prognosis (International Prognostic Index). The Local Committee of Research on Health 1301 approved this study with the number R-2022-1301-103; for the retrospective character of the study, the Ethical Committee accepted a Waiver of Informed Consent. We included 39 patients who were newly diagnosed, untreated, and without any autoimmune disease previously reported.

Any patient with Non-Hodgkin Lymphoma previously diagnosed and with established treatment, or patients with autoimmune disease, were excluded from the study.

### Statistics

The statistical analysis was divided into two phases. In the descriptive phase, to measure central tendency, it used the median value, and for the dispersing values, it used the minimum to maximum values; all proportions’ representations were expressed as percentages. The normality of the results was tested with the Shapiro–Wilks test. The Mann–Whitney U Test and cross-table Chi-square test were used in the inferential phase. A *p*-value of ≤0.05 was considered statistically significant.

For the statistical evaluation of the findings in our study, we use GraphPad Prism version 9.5 and R Studio.

## 3. Results

The median patient age was 56 years old, from 18 to 85 years old; the median weight was 67 kg, with minimum to maximum values from 47 to 100 kg; and the median height was 165 cm, with a range of 146–180 cm. The BMI median was 25 kg/m^2^, with a range from 15 to 39. Regarding the medical history of the patients, 11 (28%) subjects had hypertension, 10 (26%) had diabetes, 5 (13%) had a history of cancer, 4 (10%) had a history of kidney disease, 3 (8%) had a history of infections, and 2 (5%) had a history of alcoholism. The presence of autoantibodies was distributed as follows: 30 (77%) with autoantibodies and 9 (23%) without autoantibodies. Table 1 expresses the data:

Regarding the clinical characteristics related to staging for the NHL patients described in Table 2, we used the Ann Arbor classification system. Six (15%) patients were in stage I, 8 (21%) in stage II, 7 (18%) in stage III, and 18 (46%) in stage IV. Staging for NHL subdivides patients according to the absence or presence of disease-related symptoms: 9 (23%) were on A variation, referring to the absence of systemic symptoms; 6 (15%) were on B variation, referring to the presence of systemic symptoms; 23 (59%) were with X variation or Bulky disease; and 1 (3%) patient had splenic involvement (S). Another clinical aspect considered was the International Prognostic Index (IPI), which predicts survival to 5 years. Thus, 11 (28%) patients scored 1 point, giving them a prognosis of survival of 69%; 16 (41%) patients scored 2 points, giving them a prognosis of survival of 46%; and 12 (31%) patients scored ≥ 3, giving them a prognosis of survival of 32%.

Of the 39 patients, 30 (77%) had the presence of autoantibodies. Of these patients, 12 (40%) only present positivity to lupus anticoagulant (AL), 7 (23.3%) present positivity to AL and antinuclear antibodies (ANA), 6 (20%) present triple positivity to AL, ANA, and antineutrophil cytoplasmic antibodies (ANCA), 3 (10%) were positive to ANA and ANCA, 1 (3.3%) was positive to AL and ANCA, and 1 (3.3%) was positive just to ANA autoantibodies. This information is reflected in Table 3.

Regarding the histological subtypes obtained from the medical history, the predominant histological subtype was diffuse large B-cell lymphoma, which was identified in 23 (57%) patients. The T/NK cells subtype was identified in 4 patients (10%), mantle cell lymphoma in 2 (5%), primary central nervous system lymphoma in 2 (5%), anaplasic lymphoma in 2 (5%), Burkitt lymphoma in 1 (3%), follicular lymphoma in 1 (3%), small lymphocytes lymphoma in 1 (3%), plasmablastic lymphoma in 1 (3%), lymphoblastic lymphoma in 1 (3%), and primary mediastinum lymphoma in 1 (3%).

A comparison was made by dividing the subjects into two groups: NHL patients with and NHL patients without autoantibodies. Of the 30 (77%) NHL patients with autoantibodies, 11 (37%) were female and 19 (63%) were male. The median age for this group was 52, with a range from 18 to 85 years; the median weight was 67 kg, with minimum to maximum values from 47 to 100 kg; and the median height was 164 cm, with a range of 146–180 cm. The BMI median was 25 kg/m^2^, with a range from 15–39 kg/m^2^. Regarding the 9 (23%) patients with the absence of autoantibodies, 3 (33%) were female and 6 (67%) were male. The median age for this group was 64, with a range from 18 to 76 years; the median weight was 63 kg, with minimum to maximum values from 60 to 89 kg; and the median height was 165 cm, with a range of 153–170 cm. The BMI median of 24.9 kg/m^2^ ranges from 21.7 to 32.3 kg/m^2^. When comparing these two groups, we did not find statistical significance in these variables.

A comparison regarding the clinical characteristics related to the staging and prognosis between groups displayed in Table 4 shows the following: Of the 30 patients with the presence of autoantibodies, 13 (43%) were in stage IV, 6 (20%) were in stage III, 5 (17%) were in stage II, and 6 (20%) were in stage I. In this same group, 8 (27%) did not have systemic symptoms (A variation), 6 (20%) did have systemic symptoms (B variation), 15 (50%) had Bulky disease, and 1 (3%) had splenic involvement. Of the 9 (23%) patients without autoantibodies, 5 (56%) were in stage IV, 3 (33%) were in stage II, and 1 (11%) was in stage III. In this same group, 8 (89%) had Bulky disease and 1 (11%) did not have systemic symptoms (A variation). The comparison between groups in the early stages (I and II) and groups in the advanced stages (III and IV) was not significant (*p* = 0.55). For Ann Arbor variation due to the small sample, the comparison was made only on Bulky disease, with a *p*-value of >0.0001 with statistical significance.

From the 30 patients with autoantibodies, 7 (23%) had 1 point (low risk) on the International Prognostic Index, 12 (47%) had 2 points (intermediate risk), and 9 (30%) had 3 or more points (high risk). In the group with the absence of autoantibodies, 4 (44%) had 1 point (low risk), 2 (22%) had 2 points (intermediate risk), and 3 (34%) had 3 or more points (high risk). The proportions comparison showed a significant *p*-value of 0.003.

The following data came from the complete blood count performed before both groups initiated chemotherapy. The group that is positive to autoantibodies presented a median hemoglobin of 12.5 g/dL, with ranges from 7.9 to 15.8; hematocrit at 34.7%, with ranges from 24.5 to 51.3; median platelets of 331,000 mcL, with ranges from 64,000 to 622,000; median leukocytes of 7115 mcL, with ranges from 1690 to 39,490; and median neutrophils of 4220 mcL, with ranges from 1770 to 36,980. The group with absent autoantibodies had a median hemoglobin of 10.2 g/dL, with ranges from 8.8 to 12.8; hematocrit at 33.3%, with ranges from 27.1 to 38.3; median platelets of 314,000 mcL, with ranges from 210,000 to 413,000; median leukocytes of 5440 mcL, with ranges from 3650 to 10,770; and median neutrophils of 3900 mcL, with ranges from 2820 to 6970. In the comparison between groups, statistical significance was not found in these variables. These results are reported in Table 5.

Table 5 also describes the blood chemistry values prior to the administration of chemotherapy. It shows that in the group with autoantibodies, the patients presented a median glucose of 96 mg/dL, with a range of 60–144; urea values of 35 mg/dL, with a range of 5.5–89; and a median creatinine level of 0.74 mg/dL, with a range of 0.28–2.22. In the group of patients with the absence of autoantibodies, the median glucose was 82 mg/dL, with a range from 69 to 119 mg/dL; the median urea was 23.9 mg/dL, with minimum to maximum values from 7.9 to 36; and the median creatinine was 0.7 mg/dL, with a range of 0.54–1.12. The comparison between groups for glucose shows a *p*-value of 0.17; for urea, the comparison gave a *p*-value of 0.23; and for creatinine, the comparison was 0.77. All this shows no statistical difference between groups.

Regarding the chemotherapy treatments for the 39 patients with NHL, 31 (79%) received R-CHOP and equivalent treatments (R-COP, R-EPOCH, CHOEP, R-CNOP, CHOP, R-CO, R-CHP). In contrast, 8 (21%) patients received treatments not equivalent to R-CHOP (Dex/Met/L asparaginase, HyperC-VAD, R-MiMtx, Met/Folinic acid, R-MINE). All this is illustrated in Table 6.

The post-chemotherapy response of the 39 patients evaluated by the hematologist in charge was analyzed. In the group with autoantibodies, it was found that 12 (40%) patients had a complete response, 6 (20%) had a partial response, 1 (3%) had stable disease, 5 (17%) experienced disease progression, and 6 (20%) died. Regarding the absence of autoantibodies, 3 (33%) patients had a complete response, 3 (33%) had a partial response, 1 (12%) experienced disease progression, and 2 (22%) died.

The outcome of the patients had two categories: (a) favorable (a construct that accumulates both the complete and partial response) or (b) non-favorable (a construct accumulating stable disease, progression of the disease, and death).

When evaluating the response at the end of the administration of chemotherapy, in the group with positive autoantibodies, 18 (60%) patients had a favorable outcome and 12 (40%) patients had an unfavorable outcome. Of the patients without autoantibodies, 6 (66%) patients had a favorable outcome and 3 (34%) had an unfavorable outcome. The *p*-value for the comparison was 0.46, representing a non-statistical difference. (See Table 7).

Subsequently, when assessing the outcome of the 39 patients with Non-Hodgkin Lymphoma one year after starting the treatment described in Table 7, of the group with the presence of autoantibodies, 12 (40%) patients had a complete response, 4 (13%) patients had a partial response, 1 (3%) had stable disease, 6 (21%) had disease progression, and 7 (23%) died. Regarding the group with no presence of autoantibodies, 3 (34%) patients had a complete response, 2 (22%) had a partial response, 2 (22%) had disease progression, and 2 (22%) died.

The favorable and unfavorable outcome construct allows us to evaluate the proportions statistically. Of the patients with autoantibodies, 16 (53%) had a favorable outcome, and 14 (47%) had an unfavorable outcome. Of the patients without autoantibodies, 5 (56%) had a favorable outcome and 4 (44%) had an unfavorable outcome. The *p*-value was 0.77, which does not show significance between the outcomes and the presence of autoantibodies one year after starting the drug treatment.

Subsequently, an analysis of the 30 patients with autoantibodies compared their outcomes at the end of chemotherapy and at the end of the year of chemotherapy. Of these 30 patients, 18 (60%) had a favorable outcome at the end of chemotherapy and 12 (40%) had an unfavorable outcome. In contrast, 16 (53%) patients had a favorable outcome one year after chemotherapy and 14 (47%) patients had an unfavorable outcome. The *p*-value was 0.31, which represents non-statistical significance. (See Table 8)

In Table 8, we used the same analysis strategy for patients without autoantibodies, comparing the outcomes at the end of chemotherapy and after one year of the chemotherapy. Of these 9 patients, 6 (66%) had a favorable outcome at the end of the drug cycle and 3 (34%) had an unfavorable outcome. In contrast, 5 (56%) patients had a favorable outcome at one year of treatment and 4 (44%) patients had an unfavorable outcome. Fisher’s exact test was performed, which gave a non-significant of *p* = 0.14

## 4. Discussion

This study has sought to observe the initial characteristics in patients with NHL, mainly the frequency with or without autoantibodies, and to establish whether their presence is somehow related to the outcome of the patients.

Related to general characteristics, such as the mean age, minimum to maximum age ranges, and distribution by sex, our population is similar to what has been reported in the literature, as described by Teke et al. [8] in 2014, Lang et al. [13] in 2018, and Kungwankiattichai et al. [14] in 2020, which represent the population on the global perspective. Our population especially matches with the type of population described in the study of Hernández-Ruiz [15] in 2021, which represents patients from Mexico.

Regarding the staging and prognostic characteristics of the patients, and the distribution of the proportions of the initial stage of Ann Arbor, our results report a more significant predominance in the late stages coinciding with that reported in other studies, such as that of Bilici et al. [16] and Hernández-Ruiz [15]. Regarding the proportions of the different IPI scores of the patients in our study, they were similar to those reported in the literature, except in patients with a high IPI, including in the studies by Sehn et al. [17] and Advani et al. [18], where a higher proportion of patients with an IPI classification of high risk was observed. Nevertheless, our population has similar IPI scores to those described by Hernández-Ruiz [15].

The prevalent histological subtype was diffuse large B-cell lymphoma (DLBCL) with 57%, 10% of patients had the subtype of the T/NK cell, and the remaining 33% were composed of various histological subtypes related to B lymphocytes. This variety of subtypes of histological studies is consistent with what is reported in the literature in some review articles, such as Armitage et al. in 2017 [5], de Leval et al. in 2020 [19], and Thandra et al. [20] in 2021, just as it is consistent with studies such as the 2016 International NHL Classification Project [21] and the study made in 2021 by Hernández-Ruiz [15] in Mexico, in which they had DLBCL as the most prevalent subtype and the T/NK cell subtype as their second most prevalent subtype, which matches our findings.

The presence of autoantibodies in our study group coincides with other studies involving patients with NHL and the presence of autoantibodies [11,14,16,22,23]. We found an incidence of 77% of autoantibodies in patients with NHL at the time of diagnosis and before receiving their pharmacological cycle, which is higher than what was previously reported in the literature, which reported a prevalence ranging from 4% [16] of ANA antibodies to 41% [22] in other studies.

The difference in the prevalence of autoantibodies in patients with Non-Hodgkin Lymphoma is varied in the literature and over the years. In a study carried out in 1995, Sciarra et al. [24] detected AL and aCL at the moment of diagnosis in 9 (41%) patients with NHL, unrelated to treatment response [24]. However, they included patients with Acute Myeloid Leukemia in their population. The main difference between this study and our study is that they had a control population for antibody values, and our study is focused exclusively on patients with Non-Hodgkin Lymphoma who had not received treatment before the measurement of autoantibodies.

In 2008, Altintas et al. [25] studied 119 NHL patients and 60 Hodgkin Lymphoma (HL) patients. In the NHL patients, they reported 28.5% of positivity to ANAs, and of the patients diagnosed with HL, autoimmune diseases occurred in 6.7%. However, in our population, 57% of the patients had positivity to ANAs, and none had autoimmune diseases.

In 2012, Bilici et al. [16] reported patients with recently diagnosed NHL and HL with active disease and no disease progression. In that study, ANAs were in 5% and ANCAs were in 7%, expressing higher titers in the DLBCL subtype, and antiphospholipid antibodies were in 26% of patients [16]. Our population has a higher percentage of autoantibodies, with 30 (77%) patients with NHL; within this group, 63% are of the DLBCL subtype.

In 2014, Teke et al. [8] studied patients with HL and NHL, and they found the presence of aCL (16%) and AL (21%). However, unlike our study, they did not have patients with ANA positivity, and the patients who were ANCA-positive had a concomitant autoimmune disease. In our study, we did not consider concomitant autoimmune diseases nor the presence of aCL.

In 2020, Pal et al. [26] published a study looking for the association of antiphospholipid antibodies (APA) AL, aCL, and anti-β2 GP I with clinical parameters and outcomes in patients with NHL. They did not report significant differences in the survival between patients with and without autoantibodies [26]. Their population had autoantibodies in 23%, which contrasts with the presence of antiphospholipid in our population of 26 (87%) patients at the time of diagnosis.

The previously cited studies did not report if, at the inclusion, the patients presented chronic-degenerative comorbidities; instead, the autoimmune diseases’ presence was reported. One of the strengths of this study is the consideration of chronic-degenerative antecedents, and one of the main differences is the exclusion criteria for patients with inflammatory, rheumatic, or autoimmune diseases. In addition, another strength of this paper is the detection of the four different types of autoantibodies at the time of patient diagnosis and before receiving chemotherapy, which, unlike some studies, meant that autoantibody measurements occurred regardless of the stage.

Our study reports comprehensive medical data before the patients receive chemotherapy, giving a baseline to the status at the start of the management. Many studies contemplate more than one hematological malignancy; regardless, this study focuses only on NLH; additionally, we can provide information on the outcome of the patients not only after finishing the initial chemotherapy, but also one year after initiating treatment. This data has yet to be discussed in original articles on the Mexican population.

The following weaknesses were observed during the study: our sample size was small in comparison with other cohorts. Moreover, possibly due to an ambiguity of the clinical records and an absence of a consensus regarding relapse, refractoriness, and how to measure this variable, we did not consider this variable for statistical analysis. Another identified weakness was that we informed the autoantibodies in a dichotomous score.

## 5. Conclusions

This study presents an elevated frequency of autoantibodies (up to 77%) compared to previous reports. From this percentage of patients with an expression of autoantibodies, the majority (up to 75%) have antiphospholipid antibodies at the time of diagnosis. Our study in comparison with the international literature shows an important frequency of the presence of autoantibodies in NHL patients before beginning their treatment. This would be worth exploring in further research studies, as what might have occurred is that as a third level help facility, our consecutive cases overrepresent those expressing circulating autoantibodies referred from the second level health facility, conditioning a potential accrued sample expressing autoantibodies.

After analyzing the results, we can state that we do not find any statistical evidence that relates the presence or absence of autoantibodies with the outcome of patients with Non-Hodgkin Lymphoma, regardless of whether they are finishing the treatment or one year has passed after beginning their treatment, which helps us to provide an overall perspective and behavior of the disease.

Another characteristic worth mentioning is that more than 50% of the patients had the DLBCL subtype, which was reported in another Mexican study. In the international literature, the percentage of the DLBCL subtype does not have a substantial presence. Also, this NHL subtype exhibits a predominance of antiphospholipid antibodies in the literature.

Whether these autoantibodies represent an unspecific dysregulation of the immune system in the context of NHL should be studied. In terms of the antiphospholipid antibodies profile, its association with procoagulant activation and greater prothrombotic risk should be evaluated. Moreover, this panel of autoantibodies did not have a relationship between the presence of this characteristic and the clinical outcome of patients with NHL. We suggest expanding the autoantibodies tested on this kind of patient to seek new biomarkers that could present a relation between the administration of chemotherapy and the outcomes.

## Figures and Tables

**Table 1:** Clinical and demographic characteristics from 39 Non-Hodgkin Lymphoma patients.

Characteristic	Value
**md, minimum to maximum value**	
**Sex**	**n** (%)
**Female/Male**	14 (36)/25 (64)
**Age**	56, 18–85
**Weight**	67, 47–100
**Height**	165, 146–180
**BMI**	25, 15–39
**Clinical history**	**n** (%)
** Diabetes**	11 (28)
** Hypertension**	10 (26)
** Dyslipidemias**	5 (13)
** Others**	4 (10)
** Smoking**	3 (8)
**Alcoholism**	2 (5)
**Frequency of autoantibodies**	**n** (%)
** Positive**	30 (77)
**Negative**	9 (23)

md: median; BMI: Body Mass Index.

**Table 2:** Characteristics related to staging and prognosis in 39 Non-Hodgkin Lymphoma patients.

	n (%)
Ann Arbor classification	
I	6 (15)
II	8 (21)
III	7 (18)
IV	18 (46)
Ann Arbor staging variation	
A	9 (23)
B	6 (15)
X	23 (59)
S	1 (3)
IPI	
0–1	11 (28)
2	16 (41)
≥3	12 (31)

A: presence of systemic symptoms; B: absence of systemic symptoms; X: Bulky disease; S: splenic involvement; IPI: International Prognostic Index; 1: intermedium lower risk; 2: intermedium higher risk; ≥ 3: high risk.

**Table 3:** Positive autoantibodies in 30 NHL patients.

n = 30	AL	ANA	ANCA	aCL
12 (40%)	+	-	-	-
7 (23.3%)	+	+	-	-
6 (20%)	+	+	+	-
3 (10%)	-	+	+	-
1(3.3%)	+	-	+	-
1(3.3%)	-	+	-	-

NHL: Non-Hodgkin Lymphoma; AL: lupus anticoagulant; ANA: antinuclear antibodies; ANCA: antineutrophil cytoplasmic antibodies; aCL: anticardiolipin.

**Table 4:** Comparison of clinical characteristics from 39 Non-Hodgkin Lymphoma patients regarding staging and stratification.

	Autoantibodies		*p*
	Positive	Absent	
	n = 30	n = 9	
**Ann Arbor classification**			0.55
Early (I and II)	11 (37)	3 (33)	
Advanced (III and IV)	19 (63)	6 (67)	
**Variation**			>0.0001
A	8 (27)	1 (11)	
B	6 (20)	0	
X	15 (50)	8 (89)	
S	1 (3)	0	
**IPI**			0.0003
1	7 (23)	4 (44)	
2	14 (47)	2 (22)	
≥3	9 (30)	3 (34)	

A: without systemic symptoms; B: with systemic symptoms; X: Bulky disease; S: splenic involvement; IPI: International Prognostic Index; 1: intermedium lower risk; 2: intermedium higher risk; ≥ 3: high risk. Statistical test performed: Chi-square and Fisher’s exact test.

**Table 5:** Comparison of complete blood count and blood chemistry characteristics from 39 Non-Hodgkin Lymphoma patients before the administration of chemotherapy.

	Autoantibodies		*p*
	Positive	Absent	
	n = 30	n = 9	
**Hemoglobin; g/dL**	12.5 (7.9–15.8)	10.2 (8.8–12.8)	0.193
**Hematocrit; %**	34.7 (24.5–51.3)	33.3 (27.1–38.3)	0.439
**Platelets; mcL**	331,000 (64,000–622,000)	314,000 (210–413,000)	0.600
**Leukocytes; mcL**	7115 (1690–39,490)	5440 (3650–10,770)	0.258
**Neutrophils; mcL**	4220 (1770–36,980)	3900 (2820–6970)	0.466
**Glucose; mg/dL**	96 (60–144)	82 (69–119)	0.17
**Urea; mg/dL**	35 (5.5–89)	23.9 (7.9–36)	0.23
**Creatinine; mg/dL**	0.74 (0.28–2.2)	0.7 (0.54–1.12)	0.77

Values are expressed in medians and minimum to maximum values. Statistical test performed: Mann–Whitney U test. NHL: Non-Hodgkin Lymphoma.

**Table 6:** Chemotherapy was administered to 39 Non-Hodgkin Lymphoma patients.

Chemotherapy	n (%)
**R-CHOP and equivalent chemotherapy courses** R-COP R-EPOCH CHOEP R-CNOP CHOP R-CO R-CHP	31 (79)
**Non-equivalent R-CHOP chemotherapy courses** Dex/Met/L asparaginase HyperC-VAD R-MiMtx Met/Folinic acid R-MINE	8 (21)

R-CHOP: Rituximab, cyclophosphamide, doxorubicin, vincristine, and prednisone; R-COP: Rituximab, cyclophosphamide, vincristine, and prednisone; R-EPOCH: Rituximab, etoposide, vincristine, cyclophosphamide, and doxorubicin; R-CNOP: Rituximab, cyclophosphamide, mitoxantrone, vincristine, and prednisone; R-Mi/Met: Mitoxantrone/Methotrexate; CHOEP: Cyclophosphamide, doxorubicin, vincristine, etoposide, and prednisone; Dex/Met/L-asparaginase: Dexamethasone, methotrexate, and L-asparaginase; HyperCVAD: Cyclophosphamide, vincristine, doxorubicin, and dexamethasone; R-MINE: Rituximab, mesna, ifosfamide, mitoxantrone, and etoposide.

**Table 7:** Assessment of the outcome after finished chemotherapy and one year after starting chemotherapy from 39 Non-Hodgkin Lymphoma patients.

		Favorable Outcome	Non-Favorable Outcome	*p*
**Autoantibodies after finished chemotherapy**				
Autoantibodies n (%)	Positive	18 (60)	12 (40)	0.46
	n = 30			
	Absent	6 (66)	3 (34)	
	n = 9			
**Autoantibodies one year after starting chemotherapy**				
Autoantibodies n (%)	Positive	16 (53)	14 (47)	0.77
	n = 30			
	Absent	5 (56)	4 (44)	
	n = 9			

Statistical test performed: Fisher’s exact test.

**Table 8:** Assessment of response related to the autoantibodies after finished chemotherapy and one year after starting chemotherapy in 39 patients with Non-Hodgkin Lymphoma.

		Favorable Outcome	Non-Favorable Outcome	*p*
**Autoantibodies** (**+**)		**30** (**77%**)		
After finished chemotherapy		18 (60)	12 (40)	0.31
One year after starting chemotherapy		16 (53)	14 (47)	
**Autoantibodies** (**-**)		**9** (**23%**)		
After finished chemotherapy		6 (66)	3 (34)	0.14
One year after starting chemotherapy		5 (56)	4 (44)	

Statistical test performed: Chi-square test.

## Data Availability

Not applicable.
